# Factors That Affect Knowledge-Sharing Behaviors in Medical Imaging Departments in Cancer Centers: Systematic Review

**DOI:** 10.2196/44327

**Published:** 2023-07-12

**Authors:** Maryam Almashmoum, James Cunningham, Ohoud Alkhaldi, John Anisworth

**Affiliations:** 1 Division of Informatics, Imaging and Data Sciences School of Health Sciences Faculty of Biology, Medicine and Health, The University of Manchester Manchester United Kingdom; 2 Nuclear Medicine Department, Faisal Sultan Bin Eissa, Kuwait Cancer Control Center Kuwait City Kuwait; 3 Health Information Management and Technology Department, College of Public Health Imam Abdulrahman Bin Faisal University Dammam Saudi Arabia

**Keywords:** knowledge management, knowledge sharing, medical imaging department, radiology department, nuclear medicine department, facilitators, barriers, systematic review

## Abstract

**Background:**

Knowledge management plays a significant role in health care institutions. It consists of 4 processes: knowledge creation, knowledge capture, knowledge sharing, and knowledge application. The success of health care institutions relies on effective knowledge sharing among health care professionals, so the facilitators and barriers to knowledge sharing must be identified and understood. Medical imaging departments play a key role in cancer centers. Therefore, an understanding of the factors that affect knowledge sharing in medical imaging departments should be sought to increase patient outcomes and reduce medical errors.

**Objective:**

The purpose of this systematic review was to identify the facilitators and barriers that affect knowledge-sharing behaviors in medical imaging departments and identify the differences between medical imaging departments in general hospitals and cancer centers.

**Methods:**

We performed a systematic search in PubMed Central, EBSCOhost (CINAHL), Ovid MEDLINE, Ovid Embase, Elsevier (Scopus), ProQuest, and Clarivate (Web of Science) in December 2021. Relevant articles were identified by examining the titles and abstracts. In total, 2 reviewers independently screened the full texts of relevant papers according to the inclusion and exclusion criteria. We included qualitative, quantitative, and mixed methods studies that investigated the facilitators and barriers that affect knowledge sharing. We used the Mixed Methods Appraisal Tool to assess the quality of the included articles and narrative synthesis to report the results.

**Results:**

A total of 49 articles were selected for the full in-depth analysis, and 38 (78%) studies were included in the final review, with 1 article added from other selected databases. There were 31 facilitators and 10 barriers identified that affected knowledge-sharing practices in medical imaging departments. These facilitators were divided according to their characteristics into 3 categories: individual, departmental, and technological facilitators. The barriers that hindered knowledge sharing were divided into 4 categories: financial, administrative, technological, and geographical barriers.

**Conclusions:**

This review highlighted the factors that influenced knowledge-sharing practices in medical imaging departments in cancer centers and general hospitals. In terms of the facilitators and barriers to knowledge sharing, this study shows that these are the same in medical imaging departments, whether in general hospitals or cancer centers. Our findings can be used as guidelines for medical imaging departments to support knowledge-sharing frameworks and enhance knowledge sharing by understanding the facilitators and barriers.

## Introduction

### Background

Knowledge is an important element in the success of many institutions. It allows institutions to gain competitive advantages and aids institutional survival [1,2]. To maintain these benefits, many institutions use massive resources to implement knowledge management systems and encourage knowledge sharing among the health care professionals within those institutions. Moreover, it is considered one of the main assets in health care institutions that can be used to achieve the best patient outcomes [3]. Davenport and Prusak [4] defined knowledge as “a fluid mix of framed experience, value, contextual information, and expert insights that provides a framework for evaluating and incorporating new experiences and information.” Polanyi [5] classified knowledge into 2 categories: tacit and explicit. Explicit knowledge is easy to codify and generate in tangible forms, for example, documents, manuals, and policies [6]. In contrast, tacit knowledge is the knowledge that exists in human minds and individuals’ experiences. These experiences might be revealed through interactions with health care professionals within the workplace [7].

Health care institutions are building their own knowledge management systems to enhance the use of their own knowledge [8]. The success of any knowledge management program depends on communication among health care professionals in general and sharing knowledge among them in particular [7-9]. Health care institutions have a knowledge-sharing culture by changing health care professionals’ attitudes and behavior [8]. The concept of knowledge management is related to sharing ideas, thoughts, and experiences among health care professionals to improve health care settings [8]. In contrast, effective knowledge sharing among health care professionals depends on several facilitators, and barriers affect knowledge-sharing practices. The lack of awareness of knowledge sharing in health care institutions is the main barrier to establishing knowledge-sharing practices [9]. Moreover, according to Tetroe et al [10], knowledge sharing is essential to health care, whether in the public or private sector, and it can offer greater responsibility in health planning, making decisions, and delivering several services. From a health care point of view, knowledge sharing is a crucial instrument to ensure that the correct information gets to the right person and is used for specific purposes in the right environment at the right time [11].

Knowledge sharing among health care professionals in medical imaging departments in cancer centers plays a vital role in cancer survivorship by promoting communication among health care professionals, thus enabling them to understand cases in depth with input from professionals across different disciplines and facilitating the best interpretation of results [12]. Each cancer center has unique policies to enhance knowledge sharing among their health care professionals in a particular way without affecting patient outcomes. These policies are controlled at several points to protect patients’ privacy and help them receive appropriate treatment and make correct decisions regarding their case. Furthermore, in 2016, the National Radiotherapy Advisory Group strongly recommended that National Health Service radiography services should be increased to approximately 90% to keep up with the aging population and earlier detection of cancer cases [13]. There are several actors involved in medical imaging departments, such as physicians, oncologists, radiographers, radiologists, and nuclear medicine technologists. Technologists represent the third largest group of health care professionals [14]. In addition, approximately 60% of the health care professional workers comprise allied health professionals in the United States [15]. These allied health professionals play a crucial role in medical imaging departments and have gained plenty of knowledge in their field, either theoretical or practical, and this knowledge has to be shared among them to improve patient outcomes [16]. To improve patient care and outcomes, it is important to focus on knowledge sharing among health care professionals [17]. In medical imaging departments, knowledge sharing is complex as it involves visual patterns created using plain-text annotations and images. Therefore, knowledge sharing in medical imaging departments requires a system base to share these images, for example, the picture archiving and communication system (PACS) [18].

On the basis of previous studies on knowledge sharing, the factors that affect knowledge sharing were divided into categories: facilitators and barriers. The facilitators that enhance knowledge sharing in health care institutions were classified into 3 categories: individual, departmental, and technological facilitators. The barriers that hinder knowledge sharing were divided into 4 categories: financial, administrative, technological, and geographical barriers [19-32].

### Types of Knowledge in Medical Imaging Departments

#### Tacit Knowledge

Tacit knowledge is a vital part of human reasoning. It revolves around how humans interact with each other in their surrounding environment [33]. When explicit knowledge fails to present a full explanation of an idea, tacit knowledge can help draw a clear explanation and reach a conclusion. Moreover, it is difficult to share in its nature because of the human tendency to own their knowledge, which can give them an advantage over other peers in an institution. Tacit knowledge is exhibited in medical imaging departments as thoughts, ideas, experiences, and interpretations of results regarding specific cases [33]. Moreover, tacit knowledge is embodied in routine daily work among health care professionals everywhere, even in the hospital corridors. Furthermore, tacit knowledge is considered a lecturer’s tool, which is very important for disseminating knowledge [34]. Radiologists by nature prefer to establish contact face-to-face with each other in subgroups to share their common interests [35].

Peer-to-peer networks are considered one of the most successful ways to share tacit knowledge in medical imaging departments [33]. Storytelling is a practical way to share knowledge among health care professionals in these departments. It takes place during a medical diagnosis [34]. Teamwork meetings and conferences, whether physically or digitally, allow tacit knowledge to be a dominant type of knowledge that emerges from these gatherings [34].

#### Explicit Knowledge

Explicit knowledge is knowledge that exists in a tangible form. It is easy to generate in different forms, such as documents and policies. It exists in medical imaging departments as various documents containing information such as policies, procedure manuals, hospital protocols, and quality assurance documents for monthly records [35]. Any health care professional who takes on a role in the medical imaging department has a responsibility to know these documents and how to record them monthly. These documents are stored in an accessible place to be easily referred back to at any time [36]. Moreover, these documents are stored either manually or electronically to avoid losing them under any circumstances. These documents are updated annually when necessary [36].

Sharing explicit knowledge occurs in the medical imaging department during its workday by sharing circulars, patient requests, medical imaging, and quality control for the machines monthly.

The aim of this study was to identify facilitators and barriers that have a significant effect on knowledge-sharing practices in medical imaging departments in cancer centers. In addition, this study identified whether there are any differences between knowledge-sharing practices in medical imaging departments in general hospitals and cancer centers.

### Objectives

The first objective of this systematic review was to identify the facilitators and barriers that affect knowledge sharing among health care professionals in medical imaging departments in cancer centers. The second objective was to explore whether there are different factors in terms of facilitators and barriers that affect knowledge sharing in medical imaging departments in general hospitals versus those in cancer centers.

## Methods

### Research Questions

This study was based on the PRISMA (Preferred Reporting Items for Systematic Reviews and Meta-Analyses) 2020 statement [37]. The population, intervention, control, outcome, and study design strategy was used for a comprehensive search when resources were limited. The population, intervention, control, outcome, and study design strategy for this research is outlined in Textbox 1.

**Population, intervention, control, outcome, and study design strategy.**

**Population**

- The population of interest was any health care professionals who were working in the medical imaging departments in cancer centers or not, with the included studies reporting knowledge sharing among them (radiographers, technologists, nuclear medicine specialists, physicians, practitioners, radiologists, and nurses).

**Intervention**

- This included knowledge-sharing tools, mechanisms, and procedures that enhance knowledge-sharing practices.

**Control**

- The included studies identified facilitators that enhance knowledge-sharing practices. Moreover, the studies investigated the barriers that hinder knowledge-sharing behaviors.

**Outcome**

- The general outcome of the studies was to enhance knowledge sharing among health care professionals by identifying facilitators of and barriers to knowledge-sharing practices to improve patient outcomes and services and reduce medical mistakes.

**Study design**

- The study designs involved finding the facilitators and barriers that affect knowledge-sharing practices among health care professionals in medical imaging departments in general and particularly in cancer centers.

As a result, the following research questions were addressed in this systematic review: (1) What are the facilitators of knowledge sharing among health care professionals in medical imaging departments in general and in cancer centers in particular? (2) What are the barriers that hinder knowledge-sharing practices among health care professionals in medical imaging departments in general and in cancer centers in particular? and (3) What are the differences in factors between medical imaging departments in general hospitals and cancer centers?

### Search Strategy and Sources of Information

We searched 7 databases in December 2021: PubMed Central, EBSCOhost (CINAHL), Ovid MEDLINE, Ovid Embase, Elsevier (Scopus), ProQuest, and Clarivate (Web of Science). From ProQuest, we used 8 databases: ProQuest One Academic, ProQuest Central, Health and Medical Collection, Nursing and Allied Health Database, Healthcare Administration Database, Public Health Database, Consumer Health Database, and Materials Science Collection. The specific reason for choosing these databases was their relationship with health care institutions. The search terms were designed to capture factors that affect knowledge-sharing practices among health care professionals in medical imaging departments in cancer centers. Medical Subject Heading terms were used with the Boolean operators AND and OR to enhance the search strategy by locating the relevant studies. The search strategies for all the databases are presented in Multimedia Appendix 1.

### Eligibility Criteria

The inclusion and exclusion criteria were formulated based on the main objective of the thesis to answer the research questions.

Articles were eligible if they met the following criteria: (1) studies that examined knowledge-sharing practices; (2) studies published within the last 20 years; (3) studies conducted in medical imaging departments in cancer centers; (4) studies that investigated knowledge-sharing facilitators and barriers within medical imaging departments in general and in cancer centers in particular; and (5) qualitative, quantitative, and mixed methods designs.

Articles were excluded if they met the following criteria: (1) studies published in a language other than English; (2) meeting reports, keynotes, abstracts, books, and presentations; and (3) studies related to knowledge sharing between health care professionals and patients.

### Selection Process

**Figure 1:**
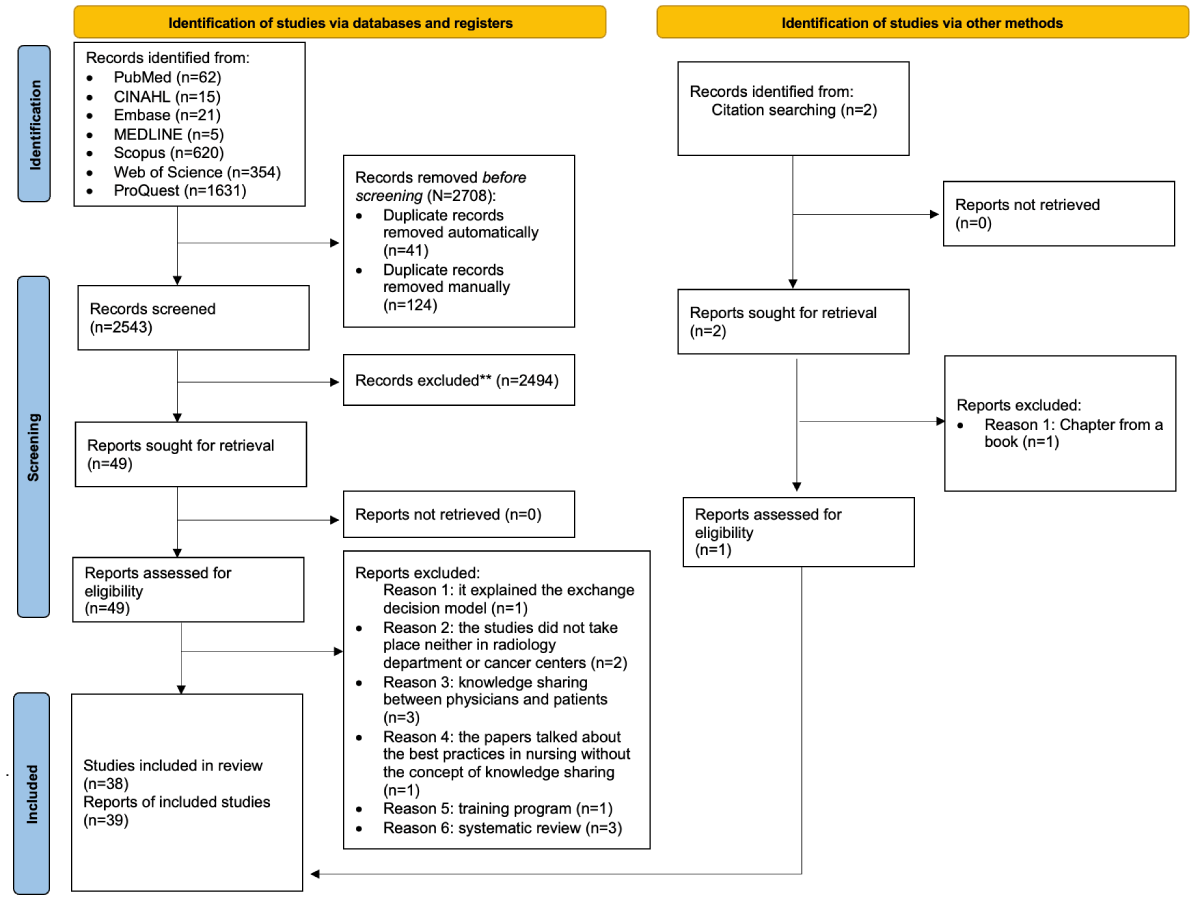
PRISMA (Preferred Reporting Items for Systematic Reviews and Meta-Analyses) 2020 statement flow diagram of the selection process for the included papers: factors, facilitators, barriers, and knowledge sharing. Medical imaging departments, cancer centers. **illegible to the inclusion and exclusion criteria.

All articles identified from the database searches were exported to EndNote Online (Clarivate Analytics), which was used for screening and eliminating any duplicates. In total, 2 reviewers (MA and OA) independently screened the titles and abstracts of all the studies. To determine whether an article should be examined in depth, the 2 reviewers assessed the article for eligibility based on the inclusion and exclusion criteria. All disagreements were resolved through discussion to make the final decision. Figure 1 shows the details of the exclusion and inclusion criteria.

### Data Collection Process

After the selection of the final studies between the 2 reviewers, the primary reviewer performed the data extraction. The data extracted from the studies included the following: names of the authors, year of publication, country, sample size, facilitators, barriers, quality of the study (based on the strong evidence of the facilitators and barriers), and main findings.

### Quality Appraisal: Risk of Bias of the Included Studies

Following the final selection of studies, the risk of bias was assessed using the Mixed Methods Appraisal Tool (MMAT) [38]. It is used for evaluation in reviews that include quantitative, qualitative, and mixed methods studies. According to the MMAT tool, assigning a single score based on the assessment is not recommended [38]. On the basis of a previous study, we used a specific statistical strategy [39] to assess the quality of each study to justify the final decision based on the inclusion and exclusion criteria. Depending on the number of criteria met, the studies were classified as high, medium, or low quality. A study was rated as high quality if all 5 MMAT criteria were met, as medium quality if 3 or 4 criteria were met, and as low quality if <2 criteria were met [39].

### Data Synthesis

This review used narrative synthesis to summarize the evidence from the final studies that were included. Narrative synthesis is useful and appropriate as this study included qualitative, quantitative, and mixed methods findings [40].

## Results

### Study Selection

The electronic search retrieved 2708 study records from 7 databases (n=62, 2.29% from PubMed Central; n=15, 0.55% from EBSCOhost [CINAHL]; n=5, 0.18% from Ovid MEDLINE; n=21, 0.78% from Ovid Embase; n=620, 22.9% from Elsevier [Scopus]; n=1631, 60.23% from ProQuest; and n=354, 13.07% from Clarivate [Web of Science]). After duplicates, which were 1.51% (41/2708) of the articles, and other articles (124/2708, 4.58%) were removed manually, of the 2708 records, 2543 (93.91%) studies remained that were assessed for title and abstract screening. In total, 1.93% (49/2543) of the studies were eligible for full-text screening, and the final number of studies included in the review was 38. In addition, 1 article was added from selected databases. A total of 39 studies were included in the review. The process and reasons for selecting these studies are shown in Figure 1.

### Characteristics of the Included Studies

Multimedia Appendix 2 [1,12,18,33-36,41-72] summarizes the characteristics of the included studies. The 39 studies included in the review were from different areas of the world: 5 (13%) were from the United States; 7 (18%) were from Canada; 6 (15%) were from Australia; 2 (5%) were from the Netherlands; 2 (5%) were from the United Kingdom; 2 (5%) were from Saudi Arabia; 2 (5%) were from Germany; and 13 (33%) were from Taiwan, Ireland, Italy, Kenya, India, Kuwait, South Africa, Sweden, France, Iran, Brazil, Finland, and Norway. Most of the included studies (26/39, 67%) presented the factors and facilitators that affect knowledge sharing among health care professionals in medical imaging departments in cancer centers. Of the 39 studies, 26 (67%) were conducted in medical imaging departments in cancer centers, whereas 13 (33%) were conducted in medical imaging departments without mentioning whether they were in cancer centers. Of the 39 studies, 21 (54%) used qualitative methods (interviews, semistructured interviews, and case studies), 14 (36%) used quantitative methods (either surveys or questionnaires), and 4 (10%) used a mixed methods approach.

The quality of the articles is shown in Multimedia Appendix 2 [1,12,18,33-36,41-72]. There were a few articles (2/39, 5%) considered weak as they related to knowledge management in general, types of knowledge, and how it is documented without any evidence of factors that affect knowledge sharing [33,34]. Therefore, those articles need to be documented in this study. For example, Barb et al [33] suggested that a successful way to share tacit knowledge is through peer-to-peer networks, whereas Zucchermaglio and Alby [34] argued that storytelling is a practical way to share tacit knowledge in medical imaging departments. Therefore, tacit knowledge has become a dominant type of knowledge in medical imaging departments [34].

### Quality of the Included Studies

Multimedia Appendix 3 [1,12,18,33-36,41-72] highlights the results of the quality assessment of the included studies. Of the 39 studies, 26 (67%) were rated as high quality as they met all 5 MMAT criteria (11/26, 42% qualitative; 12/26, 46% quantitative; and 3/26, 12% mixed methods), 11 (28%) were rated as medium quality as they met 3 or 4 of the MMAT criteria (9/11, 82% qualitative; 1/11, 9% quantitative; and 1/11, 9% mixed methods), and 2 (5%) qualitative studies were evaluated as having a low quality as they met <2 of the MMAT criteria. This review was exploratory in nature. Therefore, we decided not to exclude these studies from the final review based on the low quality regarding the MMAT criteria.

### Synthesis of the Results

#### Overview

The facilitators and barriers that affect knowledge-sharing behaviors in medical imaging departments are presented in Tables 1 and 2. We categorized the reported facilitators and barriers based on their apparent commonality according to the descriptions in previous studies [19-32]. These facilitators were divided according to their characteristics into 3 categories: individual, departmental, and technological facilitators. The barriers that hindered knowledge sharing were divided into 4 categories: financial, administrative, technological, and geographical barriers.

**Table 1:** Facilitators affecting knowledge-sharing practices in medical imaging departments (n=39).

Category and facilitator		Studies, n (%)	Reference
**Individual facilitators**			
	Positive attitude	3 (8)	Lam et al [41] Taba et al [42,43]
	Awareness	1 (3)	Al Mashmoum and Hamade [44]
	Experience	1 (3)	Taba et al [42]
	Intrinsic motivation	5 (13)	Al Mashmoum and Hamade [44] Kilsdonk et al [45] Singh et al [46] Welter et al [47] Armoogum and Buchgeister [48]
	Self-efficacy	2 (5)	Al Mashmoum and Hamade [44] Singh et al [46]
	Self-esteem	2 (5)	Taba et al [42,43]
	Trust	5 (13)	Taba et al [43] Al Mashmoum and Hamade [44] Bagayogo et al [49] Fatahi et al [50] Moilanen et al [51]
	Personality	2 (5)	Al Mashmoum and Hamade [44] Patton [52]
**Departmental facilitators**			
	Community of oncologists	1 (3)	Dicicco-Bloom and Cunningham [53]
	Community of practice	4 (10)	Armoogum and Buchgeister [48] Glicksman et al [54] Fingrut et al [55,56]
	Departmental arrangements	1 (3)	Al Mashmoum and Hamade [44]
	Leadership	6 (15)	Dorow et al [35] Lam et al [41] Al Mashmoum and Hamade [44] Moilanen et al [51] Patton [52] Lee et al [57]
	Culture	4 (10)	Patton [52] Fingrut et al [55] Taba et al [43] Mork-Knudsen et al [58]
	Interprofessional collaboration	2 (5)	Lam et al [41] Moilanen et al [51]
	Teamwork	7 (18)	Lam et al [41] Al Mashmoum and Hamade [44] Welter et al [47] Patton [52] Fingrut et al [55] Mork-Knudsen et al [58] Thingnes and Lewis [59]
	Multidisciplinary team	8 (21)	Sharma et al [1] Lam et al [41] Kilsdonk et al [45] Taba et al [43] Rankin et al [60] Kilsdonk et al [61] Kane and Luz [62] Kostaras et al [63]
	Peer review	3 (8)	Kilsdonk et al [45,61] Mathews et al [64]
	Web-based teaching	1 (3)	Stoehr et al [65]
	Web-based learning	2 (5)	Armoogum and Buchgeister [48] Shaw et al [66]
	Learning	5 (13)	Al Mashmoum and Hamade [44] Welter et al [47] Fatahi et al [50] Thingnes and Lewis [59] Obura et al [67]
	Lectures, seminars, conferences, and journal club meetings	2 (5)	Adeyelure et al [36] Armoogum and Buchgeister [48]
	Workshops and training	8 (21)	Lisy et al [12] Adeyelure et al [36] Taba et al [42] Bagayogo et al [49] Fatahi et al [50] Mork-Kundsen et al [58] Samant et al [68] Barbosa et al [69]
	Extrinsic motivation	3 (8)	Al Mashmoum and Hamade [44] Kilsdonk et al [45] Singh et al [46]
	Physician rounds	2 (5)	Adeyelure et al [36] Fatahi et al [50]
**Technological facilitators**			
	PACS^a^	8 (21)	Khajouei et al [18] Adeyelure et al [36] Taba et al [43] Al Mashmoum and Hamade [44] Welter et al [47] Fatahi et al [50] Thingnes and Lewis [59] Stoehr et al [65]
	ICT^b^	3 (8)	Adeyelure et al [36] Taba et al [43] Al Mashmoum and Hamade [44]
	Network	6 (15)	Taba et al [42,43] Armoogum and Buchgeister [48] Bagayogo et al [49] Fingrut et al [55] Addicott and Ferlie [70]
	Social media	3 (8)	Taba et al [43] Singh et al [46] Alanzi and Al-Habib [71]
	Intranet and extranet	1 (3)	Barbosa et al [69]
	Multimedia and teleradiology	2 (5)	Taba et al [43] Al-Safadi [72]
	Digital library	1 (3)	Taba et al [43]

^a^PACS: picture archiving and communication system.

^b^ICT: information and communications technology.

**Table 2:** Barriers affecting knowledge-sharing practices in medical imaging departments (n=39).

Category and barrier			Studies, n (%)		Reference
Financial barriers			1 (3)		Khajouei et al [18]
**Administrative barriers**					
	Language	3 (8)		Adeyelure et al [36] Lam et al [41] Stoehr et al [65]	
	Time	3 (8)		Adeyelure et al [36] Fatahi et al [50] Glicksman et al [54]	
	Shortage of staff	1 (3)		Fatahi et al [50]	
	Less experience	1 (3)		Stoehr et al [65]	
	Lack of transparency	1 (3)		Lam et al [41]	
**Technological barriers**					
	Network	5 (13)		Khajouei et al [18] Adeyelure et al [36] Taba et al [42,43] Bagayogo et al [49]	
	Upgrade of system	1 (3)		Khajouei et al [18]	
	Lack of equipment	1 (3)		Khajouei et al [18]	
**Geographical barriers**					
	Geographical distance	1 (3)		Armoogum and Buchgeister [48]	

#### Knowledge-Sharing Facilitators

The identified knowledge sharing facilitators in medical imaging departments were classified into 3 categories, as previously mentioned: individual, departmental, and technological factors, shown in Table 1.

#### Individual Facilitators

Individual facilitators are considered the basic factors that allow knowledge-sharing practices to exist in any institution. These facilitators depend on the health care professionals’ attitudes in medical imaging departments. There were 10 facilitators identified that were related to individual factors.

The most cited facilitator was intrinsic motivation [44-48]. Kilsdonk et al [45] illustrated that the intrinsic motivation to participate is a key concept for external peer review programs to enhance knowledge sharing among health care professionals in multidisciplinary teamwork in cancer care. It was found in a cross-sectional survey that intrinsic motivation plays an essential role in knowledge sharing among health care professionals [46]. Moreover, intrinsic motivation depends on health care professionals’ needs and interests [47]. It was one of the important facilitators that enhanced knowledge sharing among health care professionals in medical imaging departments [44,48].

In total, 13% (5/39) of the studies illustrated that trust significantly affects knowledge sharing [43,44,49-51]. To facilitate knowledge sharing among interprofessional networks, trust building among them is needed [49,51]. Trust has not only a strong influence on knowledge sharing [43,44] but also a positive influence on communication between referring clinicians and radiologists [50]. Positive attitudes play a significant role in sharing knowledge among interprofessional collaborations [41]. Positive attitudes have been found to influence social networking among breast radiologists, which in turn influences knowledge sharing [42,43]. Moreover, Taba et al [42] reported that experience was a key characteristic of individual facilitators, which affects knowledge sharing. In addition, without awareness of the importance of knowledge sharing, it will not exist in medical imaging departments [44]. Taba et al [42,43] highlighted that self-esteem is considered one of the individual factors that have a positive impact on knowledge sharing. Self-efficacy and personality were reported in 5% (2/39) of the studies, which found that they play a significant role in sharing knowledge [44,46,52].

#### Departmental Facilitators

Departmental facilitators include resources, which are provided by medical imaging departments to enhance knowledge-sharing practices among their health care professionals. There were 21% (8/39) of the studies that concentrated on multidisciplinary teams (MDTs) [1,41,43,45,60-63]. Lam et al [41] illustrated that interprofessional collaboration can improve knowledge sharing among them to increase patient outcomes. Interprofessional collaboration is defined as the process that occurs between multiple workers from different disciplines to achieve care for patients [41]. Moreover, Moilanen et al [51] showed that interprofessional collaboration plays a significant role in sharing knowledge and increasing well-being at the workplace. However, Lam et al [41] illustrated that MDTs were especially located in cancer centers to provide care for patients with cancer. These meetings were considered the best in cancer care and were very important for making decisions in Australia [1,60]. In the Netherlands, Kilsdonk et al [45,61] reported that regularly scheduled multidisciplinary meetings for sharing knowledge among medical professionals had a positive impact on making the best decisions regarding cancer cases. However, a lack of MDT meetings among health care professionals has a negative effect on patient outcomes in health care settings [53]. A survey that was conducted among MDT members showed that MDT meetings focused on sharing knowledge, collaborating, and making decisions among their specialized members. These members were from different disciplines, such as medical oncologists, radiologists, nurses, pathologists, physicians, coordinators, and radiation oncologists [62]. Radiologists reported that there were several benefits from MDT meetings, for example, gaining new knowledge and being able to discuss up-to-date information in the diagnosis of patients with cancer according to their disciplines [43]. In general, MDT meetings have positive effects on achieving consensus on diagnosis and treatment strategies based on knowledge sharing among their members [63].

There were 10% (4/39) of studies that reported that communities of practice (CoPs) have a direct impact on learning by enhancing knowledge-sharing behaviors among professional members [48,54-56]. Glicksman et al [54] highlighted that CoPs were increasingly used in the health care sector to improve patient outcomes by sharing knowledge among members. A total of 94% of interviewees reported that their experience in professional networks increased because of their involvement in the CoP [54]. Fingrut et al [55,56] showed in their study that tacit knowledge, which is the main type of knowledge, was shared during CoPs and that it is difficult to codify it. In addition, *community of oncologists* is used as a term to describe a CoP that plays a significant role in sharing information and knowledge among oncologists [53].

The importance of teamwork was reported in 18% (7/39) of the studies [41,44,47,52,55,58,59]. It has a significant role in knowledge sharing by allowing health care resources to be used in the proper way and minimizing service duplication [41]. Fingrut et al [55] reported that teamwork is very important to support collaboration with government services. Welter et al [47] illustrated that knowledge sharing takes place during teamwork to increase problem-solving strategies. According to the qualitative methods used in 5% (2/39) of the studies, interviews showed that teamwork can facilitate knowledge sharing among health care professionals in medical imaging departments [44,59]. To break the conflict among health care professionals in medical imaging departments, Patton [52] and Mork-Knudsen et al [58] reported that the role of the department is to enhance teamwork to manage workplace conflict by improving the departmental environment. Department arrangements have a positive impact on enhancing knowledge sharing by offering health care professionals the best office layout and an environment free of risk [44]. In addition, peer review is essential to improve teamwork in health care institutions and, therefore, knowledge sharing [45,61,64].

There were 21% (8/39) of studies that focused on the importance of training and workshops to support knowledge sharing [12,36,42,49,50,58,68,69]. In 2010, Armoogum and Buchgeister [48] illustrated in their survey, which took place in the radiology department, that 74% of respondents stated that seminars and journal clubs had a positive impact on supporting knowledge sharing. Adeyelure et al [36] reported that most South African health care centers play a significant role in encouraging their health care professionals to attend national and international conferences, workshops, and symposiums to facilitate knowledge sharing. Regularly attending workshops has a positive impact on knowledge sharing [50,68]. Taba et al [42] identified that breast radiology training has a positive impact on the work environment by facilitating knowledge sharing. Moreover, studies that reported training considered it a main means for knowledge-sharing accomplishment [12,69]. In addition, multidisciplinary training programs are crucial for facilitating knowledge sharing and interaction among professionals. In summary, the role of training focuses on achieving skills and maintaining a workplace environment, thereby facilitating knowledge-sharing practices among their health care professionals [58].

Web-based teaching in radiology departments played a significant role in enhancing knowledge sharing during the COVID-19 pandemic [65]. In addition, Adeyelure et al [36] and Fatahi et al [50] identified that physician rounds are considered another way of teaching and sharing knowledge among health care professionals, for example, physicians, nurses, and allied health professionals.

Learning plays a significant role in knowledge sharing, either attending physically or over the web. Web-based learning, or e-learning, enables collaborative knowledge sharing by using mobile devices or computers [66]. In 2010, Armoogum and Buchgeister [48] illustrated that web-based learning forms the shape of the body of knowledge sharing with radiology CoPs. In their studies, Welter et al [47], Al Mashmoum and Hamade [44], and Fatahi et al [50] reported that learning played a vital role in sharing tacit and explicit knowledge among health care professionals in radiology departments. The results of the survey conducted at a medical imaging department found that 95% of radiographers believed that learning and lifelong learning were important in radiography as they had a positive impact on sharing knowledge [59]. In general, learning occurs within a community to increase and support learning experiences by encouraging knowledge sharing among learners [67].

In the CoP, cultural collaboration plays a significant role in improving knowledge of outcomes of patients with cancer [55]. Taba et al [43] reported that departmental culture is very important among health care professionals in radiology departments as it has a strong impact on the workplace environment by enhancing frequently asking for further opinions. In their studies among radiographers, Barbosa et al [69] and Mork-Knudsen et al [58] showed that managers in a department have a huge responsibility to control the departmental culture by modifying it to create a strong environment for sharing health care professionals’ beliefs and thoughts and improving the practice of justification among them. This helps break the conflict among them as it encourages teamwork opportunities [52].

Leadership is the backbone of any department. Lee et al [57] reported that an empowering leader is crucial in decision-making and encourages health care professionals to share knowledge among themselves. In addition, leadership has a responsibility to support the department by allowing health care professionals to share their knowledge by building a healthy communication environment [35,44]. Furthermore, leaders play a crucial role in enhancing knowledge sharing and opening the door for creativity by breaking down conflict among health care professionals at the workplace [52]. In general, leaders have a huge responsibility to build a communication culture to enhance knowledge sharing [51].

Extrinsic motivation is a departmental facilitator that has a positive impact on knowledge sharing by providing rewards and reciprocal benefits [44-46].

#### Technological Facilitators

Technological facilitators include information and communications technologies (ICTs). The findings of 8% (3/39) of the studies indicated that ICT is considered a core facilitator for building professional social networks and enhancing work environment practices among health care professionals in medical imaging departments [36,43,44]. In total, 21% (8/39) of the studies identified the role of the PACS in knowledge-sharing practices in medical imaging departments [18,36,43,44,47,50,59,65]. Stoehr et al [65] showed that the PACS played a vital role in web-based conferences by making cases and tumors obvious in an easy way. The findings of a qualitative study revealed that the PACS has advantages in the transmission, reception, retrieval, processing, distribution, and display of medical reports and imaging from one workstation to another [43].

Several studies (5/39, 13%) found that interprofessional networks are important for facilitating knowledge sharing and improving patient outcomes [49,55]. This network could be either an intranet within a department or an extranet between one department and another [69]. In 2007, Addicott and Ferlie [70] demonstrated that networks were considered means of sharing knowledge and good practice among professionals from different disciplines and other health care institutions involved in patient care. Furthermore, a network exists in all radiology departments as it is a strong knowledge-sharing practice and positively affects the workplace [43,48]. Teleradiology and internet-based multimedia interaction play a vital role in knowledge sharing by transmitting radiographic imaging and written or spoken words from one location to another, for example, multimedia internet-based and teleradiology management systems [43,72].

Social media platforms are considered a useful tool for health care professionals [43,46]. In the results of a survey study among health care professionals, >80% of respondents stated the importance of social media in improving knowledge sharing, thereby improving decision-making [71].

The establishment of digital libraries has changed the way the radiology environment operates and the way of searching for information [43]. Interviews reported that electronic resources such as e-books and databases could support making decisions. Moreover, it is an effective source for education and solving work-related problems [43].

This section identified the facilitators that affect knowledge sharing among health care professionals in medical imaging departments. The section that follows will identify the barriers that hinder knowledge-sharing practices.

#### Knowledge-Sharing Barriers

These barriers are shown in Table 2.

Financial barriers are considered one of the main barriers that have a negative impact on knowledge-sharing behaviors, for example, the low cost to support ICT tools [18]. In addition to financial barriers, there were administrative barriers that hindered knowledge-sharing practices. Language barriers were found to be a source of reluctance on knowledge sharing among health care professionals in medical imaging departments in 8% (3/39) of the studies [36,41,65]. In addition, health care professionals were reluctant to share knowledge as they did not have enough experience to share it with others [65]. Lam et al [41] reported in their study that a lack of transparency could affect knowledge sharing because of a lack of awareness of departmental policies and visions. Furthermore, there was reduced knowledge sharing among health care professionals because of a shortage of staff [50]. Moreover, time constraints were considered a barrier that impeded knowledge sharing as health care professionals who had many tasks to achieve often did not have enough time to share knowledge [36,50,54].

Technology plays a significant role in aiding the knowledge-sharing process. However, in several studies (5/39, 13%), it was found that low-speed networks had a negative impact on knowledge sharing as most tools that support knowledge sharing require a high-speed network [18,36,42,43,49]. Khajouei et al [18] showed that the lack of equipment and support for upgrading systems affected knowledge-sharing behaviors. Finally, the distance between geographically spread health care professionals caused communication issues [48].

## Discussion

### Principal Findings

#### Overview

This study identified the factors that affect knowledge sharing in medical imaging departments in cancer centers. The analysis of the selected 39 articles revealed that medical imaging departments have several facilitators and barriers affecting the knowledge-sharing process. All those facilitators and barriers can apply to all medical imaging departments in general hospitals and cancer centers. All the selected studies (39/39, 100%) were conducted in medical imaging departments. However, 67% (26/39) were conducted in cancer centers.

#### Knowledge-Sharing Facilitators in Medical Imaging Departments in General Hospitals Versus Cancer Centers

The findings of this study revealed that all facilitators can apply to all medical imaging departments in general hospitals and cancer centers. However, some of the terminology of facilitators is different in medical imaging departments in cancer centers because of the nature of dealing with cancer cases in these centers. The differences in facilitator terminology between medical imaging departments in general hospitals and cancer centers are shown in Table 3.

**Table 3:** Facilitators that affect knowledge sharing in medical imaging departments in general hospitals versus cancer centers.

Type of facilitator	Facilitator terminology in medical imaging departments in general hospitals	Facilitator terminology in medical imaging departments in cancer centers
Individual facilitators	Trust Positive attitudes Awareness Experience Intrinsic motivation Personality Self-esteem Self-efficacy	Trust Positive attitudes Awareness Experience Intrinsic motivation Personality Self-esteem Self-efficacy
Departmental facilitators	CoPs^a^ and interprofessional collaboration Leadership Culture Teamwork Extrinsic motivation Learning and training Physician rounds Departmental arrangements	MDT^b^ and community of oncologists Leadership Culture Teamwork Extrinsic motivation Learning and training Physician rounds Departmental arrangements
Technological facilitators	ICT^c^ (PACS^d^, social media, intranet, extranet, telemedicine, and teleradiology) Network Digital library	ICT (PACS, social media, intranet, extranet, telemedicine, and teleradiology) Network Digital library

^a^CoP: community of practice.

^b^MDT: multidisciplinary team.

^c^ICT: information and communications technology.

^d^PACS: picture archiving and communication system.

First, individual facilitators play a significant role in enhancing knowledge sharing in medical imaging departments. They comprise several facilitators that are consistent in medical imaging departments be it in a cancer center or not. Trust has been proven to be an important determinant of knowledge sharing. Trust is the backbone of any relationship, so it enables knowledge sharing in medical imaging departments, especially tacit knowledge. Building trust among health care professionals who have less experience is important to enhance knowledge sharing among those who are experts in their field [49]. Fatahi et al [50] reported several actions to increase interprofessional trust, for example, face-to-face communication and phone contacts between referring clinicians and radiologists.

The importance of intrinsic motivation, which is related to knowledge sharing, could be observed in several studies (5/39, 13%). Intrinsic motivation has a direct impact on knowledge-sharing attitudes [46]. For instance, when health care professionals are not motivated to share what they have, they tend to keep the knowledge to themselves. In addition, positive attitudes are directly related to existing knowledge-sharing behaviors as they motivate health care professionals to share knowledge. The awareness of the importance of knowledge sharing among health care professionals is important to encourage them to share knowledge frequently to increase patient outcomes. Furthermore, personality is considered an individual facilitator that enhances knowledge sharing such that health care professionals who have positive attitudes tend to share knowledge with their peers. Self-efficacy and self-esteem are also important traits that motivate health care professionals to share their knowledge. In general, individual facilitators are crucial in medical imaging departments to build knowledge-sharing environments.

Second, there are departmental facilitators that enhance knowledge sharing among health care professionals in medical imaging departments. The existence of these facilitators is directly related to the success of the departmental policies. Although these facilitators are the same in all medical imaging departments, the terminology that describes CoPs in cancer centers is different. These are called *MDTs* and *community of oncologists*.

Culture has been identified as a vital departmental facilitator that enables knowledge-sharing practices. In addition, Fingrut et al [55] reported that cultural communication plays a very important role in building a CoP to improve cancer care. Culture is a powerful facilitator to share knowledge by creating a healthy environment for innovation, community, and freedom to ask questions [43]. Leadership plays a crucial role in enhancing knowledge sharing among health care professionals in medical imaging departments, for example, breaking down conflict among them by understanding them and giving them opportunities to work with each other in a healthy environment [52]. Moreover, a leader is responsible for building their trust and motivating them to share knowledge among them. The concept of empowering leadership in relation to knowledge sharing was observed in the study by Lee et al [57]. This study illustrated that empowering leadership plays a vital role in promoting knowledge-sharing behaviors among health care professionals. Administration arrangements tend to affect the transfer of both types of knowledge by offering health care professionals spaces and offices to share their knowledge in a proper way [44]. Furthermore, both intrinsic and extrinsic motivation have a positive impact on knowledge-sharing attitudes by giving health care professionals awards and bonuses for sharing knowledge [44,46].

From the analytical review of the articles, there are several communities that support knowledge sharing in medical imaging departments. In general hospitals, the most popular community is called the CoP. It has become more common throughout medical imaging departments to share knowledge, with the major goal of enhancing the quality of services [54]. In addition, interprofessional collaboration is another type of community that enhances knowledge sharing through collaboration among several professionals with different knowledge backgrounds to achieve the highest quality of care [41]. However, in cancer centers, there are several communities, for instance, the community of oncologists. Dicicco-Bloom and Cunningham [53] illustrated that the purpose of this community is to give oncologists the chance to share their knowledge regarding special cancer care to improve patient outcomes. Furthermore, there is the MDT meeting. This kind of meeting plays a significant role in sharing knowledge among professionals to make a proper decision regarding specific cancer cases that are involved in this meeting based on several requirements to select the patient in question [53]. In general, teamwork, either within a community or in a separate group, plays an obvious role in building strong knowledge-sharing behaviors in radiology departments [58].

Workshops and training such as lectures, seminars, conferences, and journal club meetings are essential to circulate tacit and explicit knowledge among health care professionals in medical imaging departments [48]. Medical imaging departments should organize these activities annually (such as conferences), monthly (such as workshops), weekly (such as lectures and journal club meetings), and daily (such as morning sessions) to create an active environment for sharing knowledge among health care professionals. Furthermore, learning in radiology centers plays an essential role in making decisions by developing radiologists’ ability to use the available tools (eg, PACS) to retrieve images to share with other colleagues [47]. During the COVID-19 pandemic, web-based learning became predominant because of social distancing. Therefore, web-based learning is the best tool to enhance knowledge sharing among health care professionals without having to consider geographical barriers.

Finally, there are technological facilitators that affect knowledge-sharing practices in medical imaging departments. These facilitators are consistent in medical imaging departments whether in general hospitals or cancer centers. They have a positive impact on knowledge sharing in medical imaging departments. The role of ICT in knowledge sharing has become very important because of the teleological revolution. The most cited type of ICT facilitator was the PACS, which is well-known in medical imaging departments. The PACS is a powerful tool that encourages knowledge sharing among health care professionals by providing them with the ability to send and receive many reports and images of different patient cases from one location to another [18]. This interaction to share knowledge can happen within a department or among different departments [36]. This type of facilitator is used only in medical imaging departments. There are 2 ways to facilitate internet-based intranets or extranets [69]. Although technological facilitators are important, high-speed networks are required to perform several tasks in a proper way. For instance, the UK National Health Service has established Managed Clinical Networks especially for cancer cases to streamline patient pathways and increase knowledge sharing among professionals who are involved in cancer care [70]. Social media is another example of a technological facilitator that is part of ICTs and enhances and facilitates formal and informal knowledge sharing in health care institutions. Social media such as Facebook, WhatsApp, blogs, and wikis has a strong impact on enhancing knowledge sharing among health care professionals in the health sector. The results of the survey by Alanzi and Al-Habib [71] showed that social media is a powerful instrument to enhance teaching that has a positive role in making decisions and solving problems. In addition, telemedicine and teleradiology play a significant role in enhancing knowledge sharing among health care professionals by sharing images of the scans among them to interpret an appropriate report [43,72]. Digital libraries are instrumental in enhancing knowledge sharing as they play a vital role in learning and problem-solving [43].

#### Knowledge-Sharing Barriers in Medical Imaging Departments in General Hospitals Versus Cancer Centers

In addition to the facilitators that affect knowledge sharing, there are several barriers that hinder knowledge-sharing practices. These barriers can apply to all medical imaging departments in general hospitals and cancer centers, as shown in Textbox 2. Financial barriers such as costs are considered one of the most predominant barriers that affect knowledge sharing [18]. The PACS, hospital information systems, and registration information systems require a large amount of money for upgrading and maintenance to work efficiently without losing patient information [18].

**Barriers that hinder knowledge sharing in medical imaging departments in general hospitals and cancer centers.**

**Financial barriers**

- Cost

**Administrative barriers**

- Language
- Time
- Shortage of staff
- Lack of transparency
- Lack of experience

**Technological barriers**

- Low speed in network
- Upgrade of the system
- Lack of equipment

**Geographical barriers**

- Geographical distance

The studies showed that there are administrative barriers that have a negative impact on knowledge-sharing behaviors, such as language barriers, time constraints, lack of experience, shortage of staff, and lack of transparency. The language barrier is the main barrier facing administration as language is the first route for health care professionals to communicate with their peers and share their knowledge [65]. Therefore, the administration should select a language that suits the majority. In addition, time constraints are a barrier that hinders knowledge sharing [54]. Insufficient time did not allow health care professionals in medical imaging departments to share their knowledge as they were busy with cases all the time. Thus, the administration should offer them free time to share their knowledge by attending meetings. Lam et al [41] illustrated that a lack of transparency impedes knowledge-sharing practices as the administration does not have a clear policy or framework to activate knowledge-sharing behaviors. Moreover, experts have a tendency to share their knowledge more than those who have less experience [65]. To avoid that, conducting educational practices is vital to encourage health care professionals to gain new experiences and keep them up-to-date.

There were several technological barriers, but the most cited one was networks. Poor networks can hinder not only knowledge-sharing practices but also health care procedures [18,36]. In addition, the lack of equipment has a negative impact on knowledge sharing. Maintaining and upgrading systems is essential to enhance knowledge sharing among health care professionals in medical imaging departments [18].

The distance between geographically separated health care professionals worldwide acts as a barrier and causes communication problems [48]. Knowledge sharing among health care professionals becomes easier when they meet without a geographical barrier or if the physical distance is not a concern. However, with the growth of web-based meetings, especially during the COVID-19 pandemic, teaching, learning, and meeting over the web are useful tools to maintain knowledge-sharing practices among health care professionals and break down these barriers.

This study identified factors that affect knowledge sharing in medical imaging departments in cancer centers and general hospitals. All facilitators and barriers can apply to medical imaging departments in general hospitals and cancer centers. However, we note that the terminology used to describe facilitators of and barriers to knowledge sharing is inconsistent across health care sectors depending on the facilitators and the nature of the work in those sectors. For example, in medical imaging departments in cancer centers, MDT meetings and communities of oncologists are considered a type of CoP. They constitute departmental facilitators and are used frequently in cancer centers [1,41,43,45,53,60-63].

The findings of this review are consistent with those of other studies on the factors that affect knowledge sharing in all health care settings [19-32]. This is presumably because this study focused on health care sectors, which have the same environment. This environment has demonstrated the interaction of tacit and explicit knowledge among health care professionals to share knowledge that depends on several factors. Although these factors have remained consistent, the PACS is only used in medical imaging departments, but the remaining factors can apply to different departments in general hospitals.

### Limitations and Strengths

There were several limitations to this study that should be acknowledged. There were 7 search engines that were used in this systematic review. Although these databases are relevant for health care publications, there is a possibility that unrelated studies were included. In addition, a few databases had a small number of results. As this study was restricted to only medical imaging departments, we could not determine whether the factors that affect knowledge sharing in those departments are the same for all departments in health care settings. Further work is required to assess this. To the best of our knowledge, there has been no previous systematic review that identified factors affecting knowledge-sharing practices among health care professionals in medical imaging departments in cancer centers.

### Conclusions

This systematic review revealed the factors that can serve as a framework for facilitating the overall knowledge-sharing process in any medical imaging department in a general hospital or cancer center. In terms of the facilitators of and barriers to knowledge sharing, this study showed that they are the same in medical imaging departments, whether in cancer centers or general hospitals. However, the terminologies might be different based on the nature of these departments. Medical imaging departments exist as part of health care services, and they have several tasks that have increased gradually because of advances in technology and imaging procedures, for instance, implementing new technologies for imaging and diagnosing patients’ conditions.

This study identified a source of knowledge for medical imaging departments and a clear understanding of facilitators and barriers that affect knowledge-sharing practices. Therefore, the managers and policy makers of medical imaging departments should be aware of these facilitators and barriers to create a framework that enhances knowledge sharing and avoids any challenges health care professionals might face regarding the knowledge-sharing process. Furthermore, it will inform them of the deficiencies in knowledge management implementation because of the lack of an effective knowledge-sharing process.

## Appendix

Multimedia Appendix 1 Results of the search strategies used in PubMed Central, EBSCOhost (CINAHL), Ovid MEDLINE, Ovid Embase, Elsevier (Scopus), ProQuest, and Clarivate (Web of Science).

Multimedia Appendix 2 Characteristics of the included studies.

Multimedia Appendix 3 Quality assessment of the included studies using the Mixed Methods Appraisal Tool (2018).

## Abbreviations

CoP: community of practice
ICT: information and communications technology
MDT: multidisciplinary team
MMAT: Mixed Methods Appraisal Tool
PACS: picture archiving and communication system
PRISMA: Preferred Reporting Items for Systematic Reviews and Meta-Analyses
