# Supplementary material for: Factors That Affect Knowledge-Sharing Behaviors in Medical Imaging Departments in Cancer Centers: Systematic Review
Source: JMIR Hum Factors. 2023 Jul 12;10:e44327. doi: 10.2196/44327 (PMC10372764; doi:10.2196/44327)
Supplement: Multimedia Appendix 1 [file humanfactors_v10i1e44327_app1.docx]

**Multimedia Appendix 1: Results** of the search strategies used in PubMed central, EbScohost (CINAHL), Ovid (Medline), Ovid (Embase), Elsevier (Scopus), ProQuest, and Clarivate (Web of Science)*.

| **Search strategy as used in Ovid MEDLINE(R) & Embase** |
| --- |
| **Part 1: terms (“Knowledge sharing”)**  1 knowledge sharing.mp.  2 knowledge transfer.mp.  3 knowledge exchange.mp.  4 knowledge distribution.mp.  5 knowledge retrieval.mp.  6 tacit knowledge.mp.  7 explicit knowledge.mp.  8 knowledge sharing process.mp.  **9 1 or 2 or 3 or 4 or 5 or 6 or 7 or 8 (Combined with ‘OR’ operator)**  **Part 2: Places (“hospitals”)**  10 medical imaging department*.mp.  11 imaging department*.mp.  12 cancer cent*.mp.  13 radiology.mp. or Radiology/  14 nuclear Medicine/  15 nuclear medicine.mp. or Nuclear Medicine/  16 radiology department*.mp.  17 radiology cent*.mp.  18 x ray department*.mp. or Radiology Department, Hospital/  19 nuclear medicine department*.mp.  20 nuclear medicine cent*.mp.  **21 10 or 11 or 12 or 13 or 14 or 15 or 16 or 17 or 18 or 19 or 20 (Combined with ‘OR’ operator)**  **Part 3: employees who are working in the medical imaging department (“doctors”)**  22 technologists.mp.  23 doctors.mp.  24 Physicians/  25 practitioners.mp.  26 radiographers.mp.  27 nuclear medicine technologists.mp.  28 nurses  29 radiologists  **30 22 or 23 or 24 or 25 or 26 or 27 or 28 or 29 (Combined with ‘OR’ operator)**  **31 9 and 21 and 30 (Final results combined with ‘AND’ operator)** |

| **Search strategy as used in PubMed** |
| --- |
| ((((((((("knowledge sharing") OR ("knowledge transfer")) OR ("knowledge exchange")) OR ("knowledge retrieval")) OR ("knowledge distribution")) OR ("tacit knowledge")) OR ("explicit knowledge")) OR ("knowledge sharing process")) AND (((((((((("medical imaging department*") OR ("imaging department*")) OR ("Cancer cent*")) OR (radiology)) OR ("radiology department*")) OR ("radiology cent*")) OR ("x-ray department*")) OR ("nuclear medicine department*")) OR ("nuclear medicine cent*")) OR ("nuclear medicine"))) AND ((((((((doctors) OR (physicians)) OR (nurses)) OR (radiographers)) OR (Radiologists)) OR (technologists)) OR ("nuclear medicine technologists")) OR (practitioners)) |

| **Search strategy as used in Elsevier (Scopus)** |
| --- |
| ALL ( ( ( ( ( ( ( ( ( ( *"knowledge sharing"* )  OR  ( *"knowledge transfer"* ) )  OR  ( *"knowledge exchange"* ) )  OR  ( *"knowledge retrieval"* ) )  OR  ( *"knowledge distribution"* ) )  OR  ( *"tacit knowledge"* ) )  OR  ( *"explicit knowledge"* ) )  OR  ( *"knowledge sharing process"* ) )  AND  ( ( ( ( ( ( ( ( ( ( *"medical imaging department*"* )  OR  ( *"imaging department*"* ) )  OR  ( *"Cancer cent*"* ) )  OR  ( *radiology* ) )  OR  ( *"radiology department*"* ) )  OR  ( *"radiology cent*"* ) )  OR  ( *"x-ray department*"* ) )  OR  ( *"nuclear medicine department*"* ) )  OR  ( *"nuclear medicine cent*"* ) )  OR  ( *"nuclear medicine"* ) ) )  AND  ( ( ( ( ( ( ( ( *doctors* )  OR  ( *physicians* ) )  OR  ( *nurses* ) )  OR  ( *radiographers* ) )  OR  ( *radiologists* ) )  OR  ( *technologists* ) )  OR  ( *"nuclear medicine technologists"* ) )  OR  ( *practitioners* ) ) ) |

| **Search strategy as used in Web of Science & search strategy as used in ProQuest (Using 8 databases: ProQuest central, ProQuest one academia, Health medical collection, Nursing and allied health databases, Health and administration databases, Public health databases, Consumer health databases, and Material science Collection)** |
| --- |
| (("knowledge sharing") OR ("knowledge transfer") OR ("knowledge exchange") OR ("knowledge retrieval") OR ("knowledge distribution") OR ("tacit knowledge") OR ("explicit knowledge") OR ("knowledge sharing process")) AND (("medical imaging department*") OR ("imaging department*") OR ("Cancer cent*") OR (radiology) OR ("radiology department*") OR ("radiology cent*") OR ("x-ray department*") OR ("nuclear medicine") OR ("nuclear medicine department*") OR ("nuclear medicine cent*")) AND ((doctors) OR (physicians) OR (nurses) OR (radiographers) OR (Radiologists) OR (technologists) OR ("nuclear medicine technologists") OR (practitioners)) |

| **Search strategy as used in Ebscohost (CINAHL PLUS)** |
| --- |
| **Part 1: terms (“Knowledge sharing”)**  S1 “knowledge sharing”  S2 “knowledge transfer”  S3 “knowledge exchange”  S4 “knowledge distribution”  S5 “knowledge retrieval”  S6 “tacit knowledge”  S7 “explicit knowledge”  S8 “knowledge sharing process”  **S9 S1 OR S2 OR S3 OR S4 OR S5 OR S6 OR S7 OR S8 (Combined with ‘OR’ operator)**  **Part 2: Places (“hospitals”)**  S10 “medical imaging department*”  S11 “imaging department*”  S12 “cancer cent*”  S13 radiology  S14 “nuclear Medicine”  S15 “radiology department*”  S16 “radiology cent*”  S17 “x ray department*”  S18 “nuclear medicine department*”  S19 “nuclear medicine cent*”  **S20 S10 OR S11 OR S12 OR S13 OR S14 OR S15 OR S16 OR S17 OR S18 OR S19 (Combined with ‘OR’ operator)**    **Part 3: employees who are working in the medical imaging department (“doctors”)**  S21technologists  S22 doctors  S23Physicians  S24 practitioners  S25 radiographers  S26 “nuclear medicine technologists”  S27 nurses  S28 radiologists  **S29 S21 OR S22 OR S23 OR S24 OR S25 OR S26 OR S27 OR S28 (Combined with ‘OR’ operator)**  **S30 S9 AND S20 AND S29 (Final results combined with ‘AND’ operator)** |

***All search strategies were limited to the English language and publication of Jan 2000 till Dec 2021.**

***It is done on December 2021**
