# Supplementary material for: Factors That Affect Knowledge-Sharing Behaviors in Medical Imaging Departments in Cancer Centers: Systematic Review
Source: JMIR Hum Factors. 2023 Jul 12;10:e44327. doi: 10.2196/44327 (PMC10372764; doi:10.2196/44327)
Supplement: Multimedia Appendix 2 [file humanfactors_v10i1e44327_app2.docx]

|  | Multimedia Appendix 2: The Characteristics of selected studies | | | | | | |
| --- | --- | --- | --- | --- | --- | --- | --- |
| No. | Author(s) Year, Country | Study type | Sample | Quality of paper | Facilitators | Barriers | Main Findings |
| 1 | (Addicott and Ferlie, 2007 [70]  UK | Qualitative (Case study) | - | Strong | Network | - | -Network is very important tool for enhancing knowledge sharing between hospitals. |
| 2 | Adeyelure et al., 2019 [36]  South Africa | Qualitative | -Not mentioned | Strong | Lectures/ seminars/ conferences/  Workshops/  Training/  Doctor rounds/PACS  ICT | Language/  Network/ Time | -There are several factors affect knowledge sharing such as ICT factors, organizational factors.  -The challenges of knowledge sharing are time constraints, technological constraints, and language barriers. |
| 3 | Alanzi & Al-Habib, 2020 [71]  Sadia Arabia | Quantitative | -78 healthcare quality healthcare professionals working in different hospitals in Saudi Arabia | Strong | Social media | - | -Social media plays important role in sharing knowledge among them. |
| 4 | Almashmoum & Hamada, 2019 [44] Kuwait | Mix methods | 149 respondents from medical imaging department | Strong | Awareness / Intrinsic motivation/ self-efficacy/  Trust/  Personality/  Departmental arrangements/  Leadership /  Teamwork/  Learning/  Extrinsic  motivation/  PACS/  ICT | - | -Face to Face communication is the main tool for sharing knowledge among them.  -More than half of the respondents have a high level of motivation to share knowledge. |
| 5 | Armoogum & Buchgeister, 2010 [48]  UK and Germany | Quantitative | - 35 respondent from medical professionals | Strong | Intrinsic motivation/  Online  Learning/  Journal club/ meetings/  Network | - | -The community of practice is very important because it enhances peer to peer interaction, and will increase healthcare professionals’ tacit knowledge.  -A lack of seminars and journal clubs has a negative impact on knowledge sharing. |
| 6 | Al-Safadi [72] Sadia Arabia, 2016 | Quantitative | -150 respondents’ physicians from a range of specialties | Strong | Multimedia/  Tele radiology | - | -Real- time interactive multimedia, such as videoconferencing has important role in sharing knowledge. |
| 7 | Bagayogo et al., 2016 [49]  Canada | Qualitative | - Not clear the sample | Medium | Trust/ Network | - | -Inter-professional network parts of daily knowledge sharing that increase patients’ outcomes |
| 8 | Barbosa et al., 2009 [69]  Brazil | Qualitative | - Observation of healthcare professionals in Cancer centre | Strong | Workshops/  Training/  Intranet/  Extranet | Network | -Intranet is important for sharing knowledge among healthcare professionals.  -Data mining is vital in the decision making process by allowing healthcare professionals to access and share knowledge. |
| 9 | Barb et al., 2005 [33] USA | Qualitative (Case Study) | - | Weak | - | - | -Knowledge sharing and representation are very important in making good decisions. |
| 10 | Dicicco-Bloom& Cunningham, 2013 [53]  USA | Qualitative | -11 primary care in radiology  -10 nurse practitioners | Strong | Community of oncologist | - | - A Community of oncologists is better at sharing knowledge among primary care clinicians.  - Information sharing plays a very important role in enhancing the quality of cancer care. |
| 11 | Dorow, 2018 [35]  France | Qualitative | - | Medium | Leadership | - | -Tacit knowledge is dominant in the radiology department.  -Knowledge sharing is not a simple process. |
| 12 | Fatahi et al.,2019 [50]  Sweden | Qualitative | -22 radiographers | Strong | Trust/ Learning/ Lectures/  Seminars/  Conferences/  Workshops/  Doctor rounds/ PACS | Time/  Shortage of staff | -Inter-professional communication with radiologist encourage knowledge sharing among them.  -There are several challenges that hinder inter-professional communication such as, time constraints and a shortage of staff. |
| 13 | Fingrut et al., 2018 [55]  Canada | Mix methods | - 5 persons are involved in the interviews | Medium | Community of Practice | - | -Oncology community of practice improves knowledge sharing, enhances inter-professional collaboration, and increase culture of partnership. |
| 14 | Fingrut et al., 2018 [56] Canada | Qualitative | -75 respondents (Physicians and allied health professionals) | Strong | Community  of practice/  Culture / team  Work/ Network | - | -Multidisciplinary team meetings focus on diagnosis, screening, and management of common diseases by sharing knowledge. |
| 15 | Glicksman et al., 2019 [54]  USA | Mix (Qualitative & Quantitative) | -18 participants from CoP | Strong | Community of practice/ | Time | -Community of practice projects were very important to improve the quality of patient care.  -Time is considered the main barrier that hinders CoP. |
| 16 | Kane & Luz, 2011 [62]  Ireland | Quantitative | -140 respondent MDT Members (radiologists, pathologists, oncologists, a chest physiotherapist, and specialist  nurse members of the team) | Strong | MDT | - | -Making decisions occurs during the sharing of information at the meeting. |
| 17 | Khajouei et al., 2018 [18]  Iran | Qualitative (Case Study) | - | Strong | PACS / | Finical barrier/  Network/  Upgrade system/  Lack of equipment | -Technical barriers are the most challenges aspects of PACS implementation. |
| 18 | Kilsdonk et al., 2016  [45]  Netherland | Qualitative (Case Study) | -Not Clear | Medium | MDT/Peer- review/  Extrinsic  motivation | - | -External peer review program to enhance knowledge sharing among healthcare professionals in the multidisciplinary teamwork in cancer care. |
| 19 | Kilsdonk et al., 2018 [61]  Netherland | Quantitative | 15 out of 26 (medical oncologist, oncology nurses) | Medium | Intrinsic motivation/  MDT/ Peer-  review | - | -Peer review is important to improve teamwork in cancer care.  -Motivation and experience are the main facilitators for sharing knowledge during peer review. |
| 20 | Kostaras et al., 2012 [63]  Canada | Quantitative | -35 team members | Medium | MDT | - | -Multidisciplinary team meetings are important in making decisions due to knowledge sharing. |
| 21 | Lam et al., 2016 [42]  Canada | Quantitative, (survey) | -124 Cancer radiotherapy | Strong | Positive attitudes/ Leadership/  Inter-professional  Collaboration/  Teamwork/ MDT/ | Language/  Lack of transparency | -The main tool for communication among oncologists, nurses, and medical physicists is face to face communication or by phone calls.  -There are several factors that affect inter-professional communication among them such as positive attitudes and teamwork. |
| 22 | Lee et al., 2019 [57] Taiwan | Quantitative | -775 medical staff | Strong | Leadership/ | - | -Leadership is crucial and requires a high level of trust.  - Leadership enhances knowledge sharing by providing a safe environment. |
| 23 | Lisy et al.,2020 [12]  Australia | Focus group, Qualitative | - | Medium | Workshops /  Training | - | -Healthcare professionals need more training to increase knowledge sharing among them. |
| 24 | Mathews et al., 2020 [64]  Canada | Qualitative | 14 technologists, radiologists, and educators from five imaging centres and an academic institution with associated medical imaging training programs | Medium | Peer- review | - | -Peer review to evaluate image quality among NM technologist will improve knowledge sharing among them. |
| 25 | Mork-Knudsen et al., 2021 [58]  Norway | Qualitative | -5 radiographers | Medium | Culture/ Teamwork/  Workshops/  Training | - | -Training and a supportive environment are the main things that need to be developed to support knowledge sharing. |
| 26 | Moilanen et al., 2020 [51]  Finland | Quantitative | -350 respondents from different medical disciplines | Strong | Leadership, Trust, Inter professional collaboration | Time | -Inter-professional collaboration has a significant role in knowledge sharing and increase well-being. |
| 27 | Obura et al., 2011 [67]  Kenya, USA | Mix methods | 10 radiology residents | Medium | Learning | - | -A community of learners requires face to face interaction in order to share knowledge. |
| 28 | Patton, 2020 [52]  USA | Qualitative | - 13 medical imaging technologies | Strong | Personality/  Leadership/  Culture/ teamwork |  | -Workplace conflict is the main barrier to Knowledge sharing, which can be broken it by emotional intelligence training. |
| 29 | Rankin et al., 2018 [60]  Australia | Quantitative | 40 respondents | Strong | MDT | - | -MDTs focus on making decisions by sharing knowledge. |
| 30 | Samant et al., 2010 [68]  Canada | Quantitative, (survey | -22 respondent in radiation oncology | Strong | Workshops/  Training | - | -Communication skills are crucial in shared decision making.  -Training and workshops are essential for enhancing communication skills. |
| 31 | Shaw et al., 2014 [66]  Australia | Qualitative (Multi phase project) | -Healthcare  Professionals | Strong | Online- learning | - | -E-Learning enhances knowledge sharing by allowing healthcare professionals to access to other resources through the internet. |
| 32 | Sharmaa et al., 2012 [1]  Australia | Qualitative (Case study) | - | Medium | MDT | - | -Multidisciplinary meetings with medical and allied health professionals are important to share information for making decisions. |
| 33 | Singh et al., 2018 [46]  India | Quantitative | - Cross-sectional survey design was adopted,102 respondents from healthcare professionals. | Strong | Intrinsic motivation/  Self-efficacy/  Extrinsic  Motivation/  Social media | - | -Intrinsic motivation, extrinsic motivation, and self-efficacy play crucial role in the sharing of knowledge. |
| 34 | Stoehr et al., 2021 [65]  31 different countries | Quantitative, (survey | 224 radiologists | Strong | Online- teaching/PACS | Language/  Less experience | -Online learning, which is the best way of to share knowledge in radiology education, became prominent after the pandemic. |
| 35 | Taba et al., 2017 [43] Australia | Qualitative | -17 Australian radiologists | Strong | Positive attitudes/  Self-esteem  /Trust/ culture MDT/ PACS/  ICT/ Network/  Social media/  Multimedia/  Teleradiology/  Digital library | Network | -Information communication technologies enhance communication among healthcare professionals, thereby enhancing knowledge sharing.  -The social network encourages learning and communicating through MDT. |
| 36 | Taba et al. 2016 [42]  Australia | Quantitative | -31 participants | Strong | Positive attitudes/ Experience/  Self-esteem/  Workshops/  Training/  Network | Network | -Self-esteem has a positive impact on social networks, thereby enhancing knowledge sharing. |
| 37 | Thingnes & Lewis, 2011 [59]  Australia | Qualitative | - 6 radiographers | Medium | Teamwork/  Learning/PACS | - | -Knowledge sharing takes place in the learning arena through interdisciplinary collaboration. |
| 38 | Welter et al., 2011 [47]  Germany | Qualitative (Case Study) | -Among radiologists | Strong | Intrinsic motivation/  Teamwork/  Learning/PACS | - | -Learning, teamwork, and social network have an impact on knowledge sharing.  -PACS plays a crucial role in enhancing learning methods, which is part of knowledge sharing. |
| 39 | Zucchermaglio & Alby, 2016 [34]  Italy | Qualitative | -Not mentioned | Weak | - | - | -Storytelling is considered a tool for sharing tacit knowledge in medical diagnostic work. |
