# Supplementary material for: Factors That Affect Knowledge-Sharing Behaviors in Medical Imaging Departments in Cancer Centers: Systematic Review
Source: JMIR Hum Factors. 2023 Jul 12;10:e44327. doi: 10.2196/44327 (PMC10372764; doi:10.2196/44327)
Supplement: Multimedia Appendix 3 [file humanfactors_v10i1e44327_app3.docx]

***Multimedia Appendix 3: Quality assessment of the included studies using the MMAT (2018)^a^**

| First Author, Year | 1. ^b^ Qualitative | | | | | 4. Quantitative Description | | | | | 5. Mixed Methods | | | | | Quality of the study |
| --- | --- | --- | --- | --- | --- | --- | --- | --- | --- | --- | --- | --- | --- | --- | --- | --- |
| References in Systematic review  Addicott, 2007 [70]  Adeyelure, 2019 [36]  Alanzi, 2020 [71]  Al mashmoum, 2019 [44]  Armoogum, 2010 [48]  Al-safadi, 2016 [72]  Bagayogo,  2016 [49]  Barbosa, 2009 [69]  Barb, 2005 [33]  Dicicco-Bloom, 2013 [53]  Dorow, 2018 [35]  Fatahi, 2019 [50]  Fingrut, 2018 [55]  Fingurt, 2018 [56]  Glicksman, 2019 [54]  Kane, 2011 [62]  Khajouei,  2019 [18]  Kilsdonk, 2015 [45]  Kilsdonk,  2014 [61]  Kostaras, 2012 [63]  Lam, 2015 [42]  Lee, 2019 [57]  Lisy, 2020 [12]  Mathews, 2021 [64]  Mork-Knudsen, 2021 [58]  Moilanen, 2020 [51]  Obura, 2011 [67]  Patton, 2020 [52]  Rankin, 2018 [60]  Samant, 2010 [68]  Shaw, 2014 [66]  Sharmaa, 2016 [1]  Singh, 2018 [46]  Stoehr, 2021 [65]  Taba, 2017 [43]  Taba, 2016 [42]  Thingnes, 2011 [59]  Welter, 2011 [47]  Zucchermaglio, 2016 [34] | **1.1^c^**  Yes  Yes  Yes  Yes  No  Yes  Yes  Yes  Yes  Yes  Yes  Yes  Yes  Yes  Yes  Yes  Yes  Yes  Yes  Yes  Yes  No | **1.2**  Yes  Yes  No  Yes  No  Yes  Yes  Yes  Yes  Yes  Yes  Yes  Yes  Yes  Yes  Yes  Yes  Yes  Yes  No  Yes  Yes | **1.3**  Yes  Yes  Yes  Yes  No  Yes  Yes  Yes  Yes  Yes  Yes  Yes  Yes  Yes  Yes  Yes  Yes  Yes  Yes  Yes  Yes  No | **1.4**  Yes  Yes  Yes  Yes  No  Yes  No  Yes  Yes  Yes  No  No  No  No  No  Yes  Yes  No  Yes  Yes  Yes  No | **1.5**  Yes  Yes  Yes  Yes  Yes  Yes  Yes  Yes  Yes  Yes  No  Yes  Yes  Yes  Yes  Yes  Yes  Yes  Yes  Yes  Yes  No | **4.1**  Yes  Yes  Yes  Yes  Yes  Yes  Yes  Yes  Yes  Yes  Yes  Yes  Yes | **4.2**  Yes  Yes  Yes  Yes  Yes  Yes  Yes  Yes  Yes  Yes  Yes  Yes  Yes | **4.3**  Yes  Yes  Yes  Yes  Yes  Yes  Yes  Yes  Yes  Yes  Yes  Yes  Yes | **4.4**  Yes  Yes  Yes  Yes  No  Yes  Yes  Yes  Yes  Yes  Yes  Yes  Yes | **4.5**  Yes  Yes  Yes  Yes  Yes  Yes  Yes  Yes  Yes  Yes  Yes  Yes  Yes | **5.1**  Yes  Yes  Yes  Yes | **5.2**  Yes  Yes  Yes  Yes | **5.3**  Yes  Yes  Yes  Yes | **5.4**  Yes  No  Yes  Yes | **5.5**  Yes  Yes  Yes  Yes | High  High  High  High  High  High  Medium  High  Low  High  Medium  High  High  Medium  High  High  High  Medium  Medium  Medium  High  High  Medium  Medium  Medium  High  High  High  High  High  High  Medium  High  High  High  High  Medium  High  Low |

^a^MMAT: Mixed Methods Appraisal Tool

^b^1., 4., 5.: Sections of the MMAT to evaluate studies

^c^1.1-5.5: items in each of the MMAT sections used to evaluate the qualitative quantitative^,^ and mixed methods study.

*Note: all previous studies answered ‘Yes’ to the questions that related to the screening of the MMAT:

S.1. Are there clear research questions?

S.2. Do the collected data allow to address the research questions?
